# Procrastination, depression and anxiety symptoms in university students: a three-wave longitudinal study on the mediating role of perceived stress

**DOI:** 10.1186/s40359-024-01761-2

**Published:** 2024-05-16

**Authors:** Anna Jochmann, Burkhard Gusy, Tino Lesener, Christine Wolter

**Affiliations:** Division of Prevention and Psychosocial Health Research, Department of Education and Psychology, Freie Universität Berlin, Habelschwerdter Allee 45, 14195 Berlin, Germany

**Keywords:** Procrastination, Stress, Anxiety, Depression, Student health, Longitudinal study

## Abstract

**Background:**

It is generally assumed that procrastination leads to negative consequences. However, evidence for negative consequences of procrastination is still limited and it is also unclear by which mechanisms they are mediated. Therefore, the aim of our study was to examine the harmful consequences of procrastination on students’ stress and mental health. We selected the procrastination-health model as our theoretical foundation and tried to evaluate the model’s assumption that trait procrastination leads to (chronic) disease via (chronic) stress in a temporal perspective. We chose depression and anxiety symptoms as indicators for (chronic) disease and hypothesized that procrastination leads to perceived stress over time, that perceived stress leads to depression and anxiety symptoms over time, and that procrastination leads to depression and anxiety symptoms over time, mediated by perceived stress.

**Methods:**

To examine these relationships properly, we collected longitudinal data from 392 university students at three occasions over a one-year period and analyzed the data using autoregressive time-lagged panel models.

**Results:**

Procrastination did lead to depression and anxiety symptoms over time. However, perceived stress was not a mediator of this effect. Procrastination did not lead to perceived stress over time, nor did perceived stress lead to depression and anxiety symptoms over time.

**Conclusions:**

We could not confirm that trait procrastination leads to (chronic) disease via (chronic) stress, as assumed in the procrastination-health model. Nonetheless, our study demonstrated that procrastination can have a detrimental effect on mental health. Further health outcomes and possible mediators should be explored in future studies.

**Supplementary Information:**

The online version contains supplementary material available at 10.1186/s40359-024-01761-2.

## Introduction

“Due tomorrow? Do tomorrow.”, might be said by someone who has a tendency to postpone tasks until the last minute. But can we enjoy today knowing about the unfinished task and tomorrow’s deadline? Or do we feel guilty for postponing a task yet again? Do we get stressed out because we have little time left to complete it? Almost everyone has procrastinated at some point when it came to completing unpleasant tasks, such as mowing the lawn, doing the taxes, or preparing for exams. Some tend to procrastinate more frequently and in all areas of life, while others are less inclined to do so. Procrastination is common across a wide range of nationalities, as well as socioeconomic and educational backgrounds [1]. Over the last fifteen years, there has been a massive increase in research on procrastination [2]. Oftentimes, research focuses on better understanding the phenomenon of procrastination and finding out why someone procrastinates in order to be able to intervene. Similarly, the internet is filled with self-help guides that promise a way to overcome procrastination. But why do people seek help for their procrastination? Until now, not much research has been conducted on the negative consequences procrastination could have on health and well-being. Therefore, in the following article we examine the effect of procrastination on mental health over time and stress as a possible facilitator of this relationship on the basis of the procrastination-health model by Sirois et al. [3].

### Procrastination and its negative consequences

Procrastination can be defined as the tendency to voluntarily and irrationally delay intended activities despite expecting negative consequences as a result of the delay [4, 5]. It has been observed in a variety of groups across the lifespan, such as students, teachers, and workers [1]. For example, some students tend to regularly delay preparing for exams and writing essays until the last minute, even if this results in time pressure or lower grades. Procrastination must be distinguished from strategic delay [4, 6]. Delaying a task is considered strategic when other tasks are more important or when more resources are needed before the task can be completed. While strategic delay is viewed as functional and adaptive, procrastination is classified as dysfunctional. Procrastination is predominantly viewed as the result of a self-regulatory failure [7]. It can be understood as a trait, that is, as a cross-situational and time-stable behavioral disposition [8]. Thus, it is assumed that procrastinators chronically delay tasks that they experience as unpleasant or difficult [9]. Approximately 20 to 30% of adults have been found to procrastinate chronically [10–12]. Prevalence estimates for students are similar [13]. It is believed that students do not procrastinate more often than other groups. However, it is easy to examine procrastination in students because working on study tasks requires a high degree of self-organization and time management [14].

It is generally assumed that procrastination leads to negative consequences [4]. Negative consequences are even part of the definition of procrastination. Research indicates that procrastination is linked to lower academic performance [15], health impairment (e.g., stress [16], physical symptoms [17], depression and anxiety symptoms [18]), and poor health-related behavior (e.g., heavier alcohol consumption [19]). However, most studies targeting consequences of procrastination are cross-sectional [4]. For that reason, it often remains unclear whether an examined outcome is a consequence or an antecedent of procrastination, or whether a reciprocal relationship between procrastination and the examined outcome can be assumed. Additionally, regarding negative consequences of procrastination on health, it is still largely unknown by which mechanisms they are mediated. Uncovering such mediators would be helpful in developing interventions that can prevent negative health consequences of procrastination.

### The procrastination-health model

**Fig. 1:**
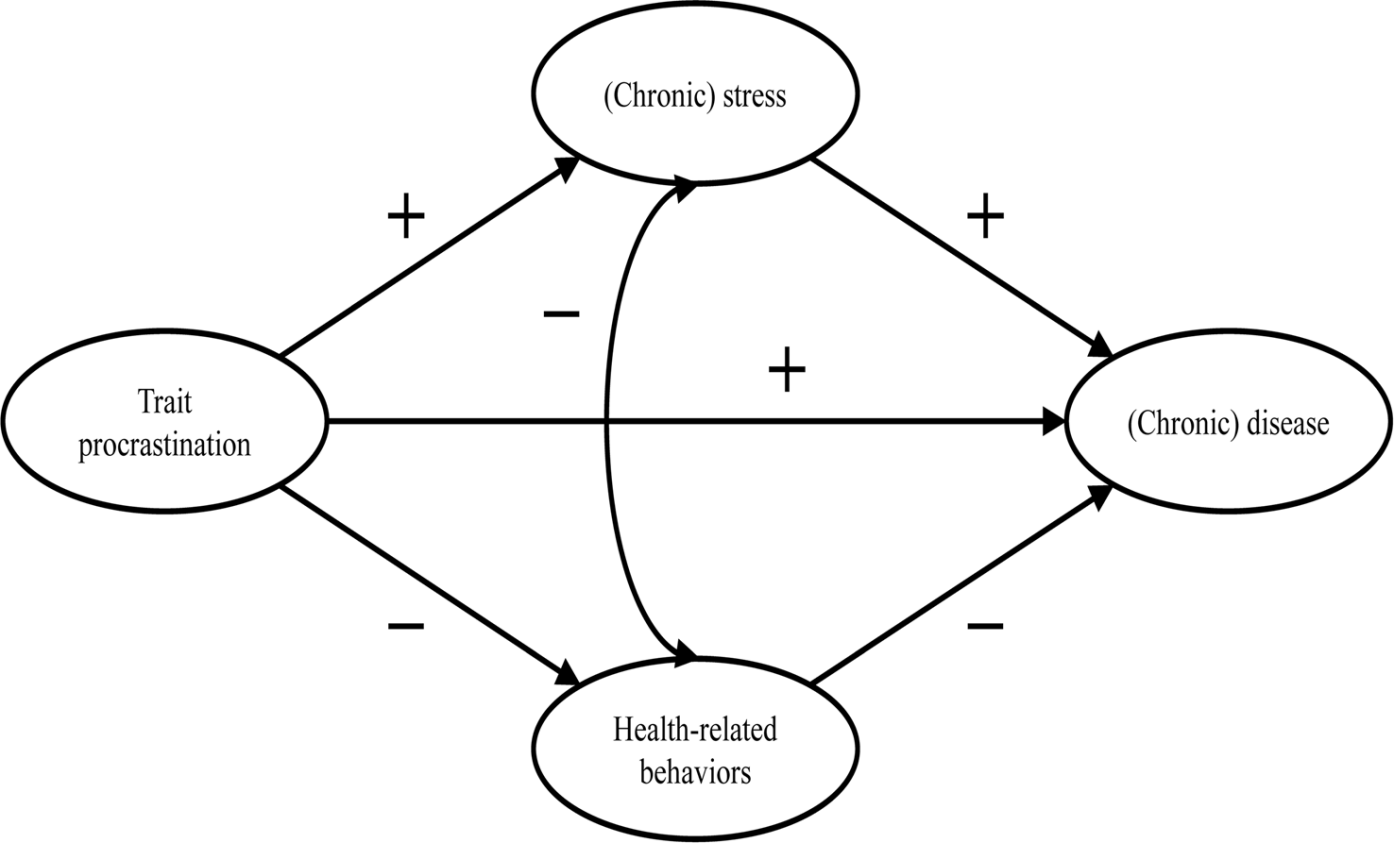
Adopted from the procrastination-health model by Sirois [9]

The first and only model that exclusively focuses on the effect of procrastination on health and the mediators of this effect is the procrastination-health model [3, 9, 17]. Sirois [9] postulates three pathways: An immediate effect of trait procrastination on (chronic) disease and two mediated pathways (see Fig. 1).

The immediate effect is not further explained. Research suggests that procrastination creates negative feelings, such as shame, guilt, regret, and anger [20–22]. The described feelings could have a detrimental effect on mental health [23–25].

The first mediated pathway leads from trait procrastination to (chronic) disease via (chronic) stress. Sirois [9] assumes that procrastination creates stress because procrastinators are constantly aware of the fact that they still have many tasks to complete. Stress activates the hypothalamic-pituitary-adrenocortical (HPA) system, increases autonomic nervous system arousal, and weakens the immune system, which in turn contributes to the development of diseases. Sirois [9] distinguishes between short-term and long-term effects of procrastination on health mediated by stress. She believes that, in the short term, single incidents of procrastination cause acute stress, which leads to acute health problems, such as infections or headaches. In the long term, chronic procrastination, as you would expect with trait procrastination, causes chronic stress, which leads to chronic diseases over time. There is some evidence in support of the stress-related pathway, particularly regarding short-term effects [3, 17, 26–28]. However, as we mentioned above, most of these studies are cross-sectional. Therefore, the causal direction of these effects remains unclear. To our knowledge, long-term effects of trait procrastination on (chronic) disease mediated by (chronic) stress have not yet been investigated.

The second mediated pathway leads from trait procrastination to (chronic) disease via poor health-related behavior. According to Sirois [9], procrastinators form lower intentions to carry out health-promoting behavior or to refrain from health-damaging behavior because they have a low self-efficacy of being able to care for their own health. In addition, they lack the far-sighted view that the effects of health-related behavior only become apparent in the long term. For the same reason, Sirois [9] believes that there are no short-term, but only long-term effects of procrastination on health mediated by poor health-related behavior. For example, an unhealthy diet leads to diabetes over time. The findings of studies examining the behavioral pathway are inconclusive [3, 17, 26, 28]. Furthermore, since most of these studies are cross-sectional, they are not suitable for uncovering long-term effects of trait procrastination on (chronic) disease mediated by poor health-related behavior.

## Our study

In summary, previous research on the two mediated pathways of the procrastination-health model mainly found support for the role of (chronic) stress in the relationship between trait procrastination and (chronic) disease. However, only short-term effects have been investigated so far. Moreover, longitudinal studies are needed to be able to assess the causal direction of the relationship between trait procrastination, (chronic) stress, and (chronic) disease. Consequently, our study is the first to examine long-term effects of trait procrastination on (chronic) disease mediated by (chronic) stress, using a longitudinal design. (Chronic) disease could be measured by a variety of different indicators (e.g., physical symptoms, diabetes, or coronary heart disease). We choose depression and anxiety symptoms as indicators for (chronic) disease because they signal mental health complaints before they manifest as (chronic) diseases. Additionally, depression and anxiety symptoms are two of the most common mental health complaints among students [29, 30] and procrastination has been shown to be a significant predictor of depression and anxiety symptoms [18, 31–34]. Until now, the stress-related pathway of the procrastination-health model with depression and anxiety symptoms as the health outcome has only been analyzed in one cross-sectional study that confirmed the predictions of the model [35].

The aim of our study is to evaluate some of the key assumptions of the procrastination-health model, particularly the relationships between trait procrastination, (chronic) stress, and (chronic) disease over time, surveyed in the following analysis using depression and anxiety symptoms.

**Fig. 2:**
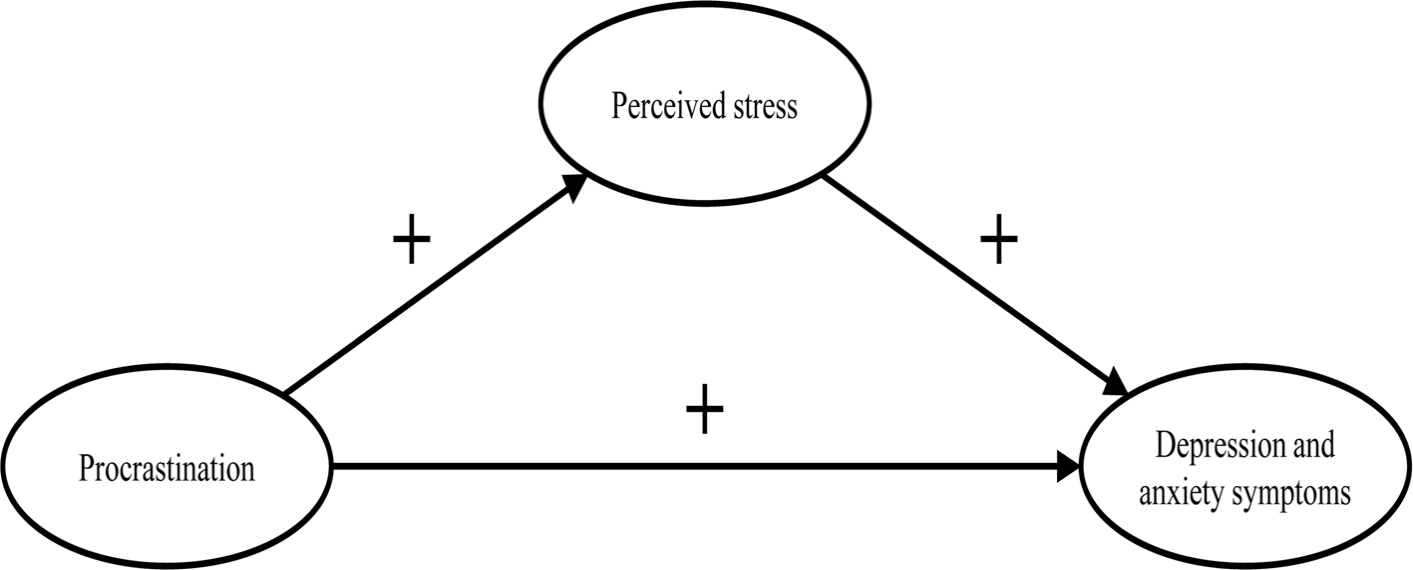
The section of the procrastination-health model we examined

In line with the key assumptions of the procrastination-health model, we postulate (see Fig. 2):

1. Procrastination leads to perceived stress over time.
2. Perceived stress leads to depression and anxiety symptoms over time.
3. Procrastination leads to depression and anxiety symptoms over time, mediated by perceived stress.

## Materials and methods

### Sample

Our study was part of a health monitoring at a large German university^1^. Ethical approval for our study was granted by the Ethics Committee of the university’s Department of Education and Psychology. We collected the initial data in 2019. Two occasions followed, each at an interval of six months. In January 2019, we sent out 33,267 invitations to student e-mail addresses. Before beginning the survey, students provided their written informed consent to participate in our study. 3,420 students took part at the first occasion (T1; 10% response rate). Of these, 862 participated at the second (T2) and 392 at the third occasion (T3). In order to test whether dropout was selective, we compared sociodemographic and study specific characteristics (age, gender, academic semester, number of assessments/exams) as well as behavior and health-related variables (procrastination, perceived stress, depression and anxiety symptoms) between the participants of the first wave (*n* = 3,420) and those who participated three times (*n* = 392). Results from independent-samples t-tests and chi-square analysis showed no significant differences regarding sociodemographic and study specific characteristics (see Additional file 1: Table S1 and S2). Regarding behavior and health-related variables, independent-samples t-tests revealed a significant difference in procrastination between the two groups (*t*(3,409) = 2.08, *p* < .05). The mean score of procrastination was lower in the group that participated in all three waves.

The mean age of the longitudinal respondents was 24.1 years (*SD* = 5.5 years), the youngest participants were 17 years old, the oldest one was 59 years old. The majority of participants was female (74.0%), 7 participants identified neither as male nor as female (1.8%). The respondents were on average enrolled in the third year of studying (*M* = 3.9; *SD* = 2.3). On average, the students worked about 31.2 h (*SD* = 14.1) per week for their studies, and an additional 8.5 h (*SD* = 8.5) for their (part-time) jobs. The average income was €851 (*SD* = 406), and 4.9% of the students had at least one child. The students were mostly enrolled in philosophy and humanities (16.5%), education and psychology (15.8%), biology, chemistry, and pharmacy (12.5%), political and social sciences (10.6%), veterinary medicine (8.9%), and mathematics and computer science (7.7%).

### Measures

We only used established and well evaluated instruments for our analyses.

#### Procrastination

We adopted the short form of the Procrastination Questionnaire for Students (PFS-4) [36] to measure procrastination. The PFS-4 assesses procrastination at university as a largely stable behavioral disposition across situations, that is, as a trait. The questionnaire consists of four items (e.g., I put off starting tasks until the last moment.). Each item was rated on a 5-point scale ((almost) never = 1 to (almost) always = 5) for the last two weeks. All items were averaged, with higher scores indicating a greater tendency to procrastinate. The PFS-4 has been proven to be reliable and valid, showing very high correlations with other established trait procrastination scales, for example, with the German short form of the General Procrastination Scale [37, 38]. We also proved the scale to be one-dimensional in a factor analysis, with a Cronbach’s alpha of 0.90.

#### Perceived stress

The Heidelberger Stress Index (HEI-STRESS) [39] is a three-item measure of current perceived stress due to studying as well as in life in general. For the first item, respondents enter a number between 0 (not stressed at all) and 100 (completely stressed) to indicate how stressed their studies have made them feel over the last four weeks. For the second and third item, respondents rate on a 5-point scale how often they feel “stressed and tense” and as how stressful they would describe their life at the moment. We transformed the second and third item to match the range of the first item before we averaged all items into a single score with higher values indicating greater perceived stress. We proved the scale to be one-dimensional and Cronbach’s alpha for our study was 0.86.

#### Depression and anxiety symptoms

We used the Patient Health Questionnaire-4 (PHQ-4) [40], a short form of the Patient Health Questionnaire [41] with four items, to measure depression and anxiety symptoms. The PHQ-4 contains two items from the Patient Health Questionnaire-2 (PHQ-2) [42] and the Generalized Anxiety Disorder Scale-2 (GAD-2) [43], respectively. It is a well-established screening scale designed to assess the core criteria of major depressive disorder (PHQ-2) and generalized anxiety disorder (GAD-2) according to the Diagnostic and Statistical Manual of Mental Disorders, Fifth Edition (DSM-5). However, it was shown that the GAD-2 is also appropriate for screening other anxiety disorders. According to Kroenke et al. [40], the PHQ-4 can be used to assess a person’s symptom burden and impairment. We asked the participants to rate how often they have been bothered over the last two weeks by problems, such as “Little interest or pleasure in doing things”. Response options were 0 = not at all, 1 = several days, 2 = more than half the days, and 3 = nearly every day. Calculated as the sum of the four items, the total scores range from 0 to 12 with higher scores indicating more frequent depression and anxiety symptoms. The total scores can be categorized as none-to-minimal (0–2), mild (3–5), moderate (6–8), and severe (9–12) depression and anxiety symptoms. The PHQ-4 was shown to be reliable and valid [40, 44, 45]. We also proved the scale to be one-dimensional in a factor analysis, with a Cronbach’s alpha of 0.86.

### Data analysis

To test our hypotheses, we performed structural equation modelling (SEM) using R (Version 4.1.1) with the package lavaan. All items were standardized (*M* = 0, *SD* = 1). Due to the non-normality of some study variables and a sufficiently large sample size of *N* near to 400 [46], we used robust maximum likelihood estimation (MLR) for all model estimations. As recommended by Hu and Bentler [47], we assessed the models’ goodness of fit by chi-square test statistic, root mean square error of approximation (RMSEA), standardized root mean square residual (SRMR), Tucker-Lewis index (TLI), and comparative fit index (CFI). A non-significant chi-square indicates good model fit. Since chi-square is sensitive to sample size, we also evaluated fit indices less sensitive to the number of observations. RMSEA and SRMR values of 0.05 or lower as well as TLI and CFI values of 0.97 or higher indicate good model fit. RMSEA values of 0.08 or lower, SRMR values of 0.10 or lower, as well as TLI and CFI values of 0.95 or higher indicate acceptable model fit [48, 49]. First, we conducted confirmatory factor analysis for the first occasion, defining three factors that correspond to the measures of procrastination, perceived stress, and depression and anxiety symptoms. Next, we tested for measurements invariance over time and specified the measurement model, before testing our hypotheses.

#### Measurement invariance over time

To test for measurement invariance over time, we defined one latent variable for each of the three occasions, corresponding to the measures of procrastination, perceived stress, and depression and anxiety symptoms, respectively. As recommended by Geiser and colleagues [50], the links between indicators and factors (i.e., factor loadings and intercepts) should be equal over measurement occasions; therefore, we added indicator specific factors. A first and least stringent step of testing measurement invariance is configural invariance (M_CI_). It was examined whether the included constructs (procrastination, perceived stress, depression and anxiety symptoms) have the same pattern of free and fixed loadings over time. This means that the assignment of the indicators to the three latent factors over time is supported by the underlying data. If configural invariance was supported, restrictions for the next step of testing measurement invariance (metric or weak invariance; M_MI_) were added. This means that each item contributes to the latent construct to a similar degree over time. Metric invariance was tested by constraining the factor loadings of the constructs over time. The next step of testing measurement invariance (scalar or strong invariance; M_SI_) consisted of checking whether mean differences in the latent construct capture all mean differences in the shared variance of the items. Scalar invariance was tested by constraining the item intercepts over time. The constraints applied in the metric invariance model were retained [51]. For the last step of testing measurement invariance (residual or strict invariance; M_RI_), the residual variables were also set equal over time. If residual invariance is supported, differences in the observed variables can exclusively be attributed to differences in the variances of the latent variables.

We used the Satorra-Bentler chi-square difference test to evaluate the superiority of a more stringent model [52]. We assumed the model with the largest number of invariance restrictions – which still has an acceptable fit and no substantial deterioration of the chi-square value – to be the final model [53]. Following previous recommendations, we considered a decrease in CFI of 0.01 and an increase in RMSEA of 0.015 as unacceptable to establish measurement invariance [54]. If a more stringent model had a significant worse chi-square value, but the model fit was still acceptable and the deterioration in model fit fell within the change criteria recommended for CFI and RMSEA values, we still considered the more stringent model to be superior.

#### Hypotheses testing

As recommended by Dormann et al. [55], we applied autoregressive time-lagged panel models to test our hypotheses. In the first step, we specified a model (M_0_) that only included the stabilities of the three variables (procrastination, perceived stress, depression and anxiety symptoms) over time. In the next step (M_1_), we added the time-lagged effects from procrastination (T1) to perceived stress (T2) and from procrastination (T2) to perceived stress (T3) as well as from perceived stress (T1) to depression and anxiety symptoms (T2) and from perceived stress (T2) to depression and anxiety symptoms (T3). Additionally, we included a direct path from procrastination (T1) to depression and anxiety symptoms (T3). If this path becomes significant, we can assume a partial mediation [55]. Otherwise, we can assume a full mediation. We compared these nested models using the Satorra-Bentler chi-square difference test and the Akaike information criterion (AIC). The chi-square difference value should either be non-significant, indicating that the proposed model including our hypotheses (M_1_) does not have a significant worse model fit than the model including only stabilities (M_0_), or, if significant, it should be in the direction that M_1_ fits the data better than M_0_. Regarding the AIC, M_1_ should have a lower value than M_0_.

## Results

Table 1 displays the means, standard deviations, internal consistencies (Cronbach’s alpha), and stabilities (correlations) of all study variables. The alpha values of procrastination, perceived stress, and depression and anxiety symptoms are classified as good (> 0.80) [56]. The correlation matrix of the manifest variables used for the analyses can be found in the Additional file 1: Table S3.

We observed the highest test-retest reliabilities for procrastination (*r* ≥ .74). The test-retest reliabilities for depression and anxiety symptoms (*r* ≥ .64) and for perceived stress (*r* ≥ .54) were a bit lower (see Table 1). The pattern of correlations shows a medium to large but positive relationship between procrastination and depression and anxiety symptoms [57, 58]. The association between procrastination and perceived stress was small, the one between perceived stress and depression and anxiety symptoms very large (see Table 1).

**Table 1:** Descriptive statistics of all study variables (*n* = 392)

		M	SD	1	2	3	4	5	6	7	8	9
1	Procrastination T1	3.15	1.07	(0.90)								
2	Procrastination T2	3.19	1.04	0.74***	(0.91)							
3	Procrastination T3	3.19	1.12	0.74***	0.76***	(0.93)						
4	Perceived stress T1	64.25	20.62	0.07	0.06	0.14**	(0.86)					
5	Perceived stress T2	64.96	19.94	0.05	0.07	0.13*	0.55***	(0.85)				
6	Perceived stress T3	65.57	19.93	0.12*	0.12*	0.20***	0.54***	0.60***	(0.85)			
7	Depression and anxiety symptoms T1	2.21	0.84	0.26***	0.25***	0.30***	0.56***	0.39***	0.42***	(0.86)		
8	Depression and anxiety symptoms T2	2.11	0.84	0.17***	0.23***	0.25***	0.49***	0.59***	0.50***	0.69***	(0.87)	
9	Depression and anxiety symptoms T3	2.13	0.86	0.26***	0.31***	0.38***	0.43***	0.43***	0.62***	0.64***	0.69***	(0.88)

*Note* Cronbach’s alpha in parentheses. T1 = time 1; T2 = time 2; T3 = time 3. * *p* < .05. ** *p* < .01. *** *p* < .001

Confirmatory factor analysis showed an acceptable to good fit (x^2^ (41) = 118.618, *p* < .001; SRMR = 0.042; RMSEA = 0.071; TLI = 0.95; CFI = 0.97). When testing for measurement invariance over time for each construct, the residual invariance models with indicator specific factors provided good fit to the data (M_RI_; see Table 2), suggesting that differences in the observed variables can exclusively be attributed to differences of the latent variables. We then specified and tested the measurement model of the latent constructs prior to model testing based on the items of procrastination, perceived stress, and depression and anxiety symptoms. The measurement model fitted the data well (M_M_; see Table 3). All items loaded solidly on their respective factors (0.791 ≤ β ≤ 0.987; *p* < .001).

**Table 2:** Comparison of configural, metric, scalar, and residual invariance models over time

Model	x^2^ (df)	*p*	SRMR	RMSEA	TLI	CFI	Ref. Model	∆x^2^ (df)	*p*
*Procrastination*									
M_CI_: configural	57.929 (45)	> 0.05	0.018	0.027	1.00	1.00			
M_MI_: metric	62.595 (51)	> 0.05	0.023	0.024	1.00	1.00	M_CI_	3.93 (6)	> 0.05
M_SI_: scalar	62.962 (57)	> 0.05	0.023	0.017	1.00	1.00	M_MI_	0.04 (6)	> 0.05
M_RI_: residual	88.249 (65)	< 0.05	0.022	0.031	0.99	0.99	M_SI_	23.76 (8)	< 0.01
*Perceived stress*									
M_CI_: configural	28.890 (21)	> 0.05	0.024	0.034	0.99	0.99			
M_MI_: metric	39.027 (25)	< 0.05	0.039	0.042	0.98	0.99	M_CI_	11.05 (4)	< 0.05
M_SI_: scalar	43.851 (29)	< 0.05	0.040	0.040	0.99	0.99	M_MI_	4.74 (4)	> 0.05
M_RI_: residual	47.400 (35)	> 0.05	0.040	0.033	0.99	0.99	M_SI_	4.60 (6)	> 0.05
*Depression and anxiety symptoms*									
M_CI_: configural	68.253 (45)	< 0.05	0.035	0.037	0.99	0.99			
M_MI_: metric	72.781 (51)	< 0.05	0.037	0.033	0.99	0.99	M_CI_	3.25 (6)	> 0.05
M_SI_: scalar	73.812 (57)	> 0.05	0.037	0.028	0.99	0.99	M_MI_	0.13 (6)	> 0.05
M_RI_: residual	81.455 (65)	> 0.05	0.037	0.026	0.99	0.99	M_SI_	7.83 (8)	> 0.05

*Note* x^2^ = chi-square value; *df* = degrees of freedom; SRMR = standardized root mean square residual; RMSEA = root mean square error of approximation; TLI = Tucker-Lewis index; CFI = comparative fit index; AIC = Akaike information criterion; ∆x^2^ = Satorra-Bentler chi-square difference test [52]

To test our hypotheses, we analyzed the two models described in the methods section.

The fit of the stability model (M_0_) was acceptable (see Table 3). Procrastination was stable over time, with stabilities above 0.82. The stabilities of perceived stress as well as depression and anxiety symptoms were somewhat lower, ranging from 0.559 (T1 -> T2) to 0.696 (T2 -> T3) for perceived stress and from 0.713 (T2 -> T3) to 0.770 (T1 -> T2) for depression and anxiety symptoms, respectively.

**Fig. 3:**
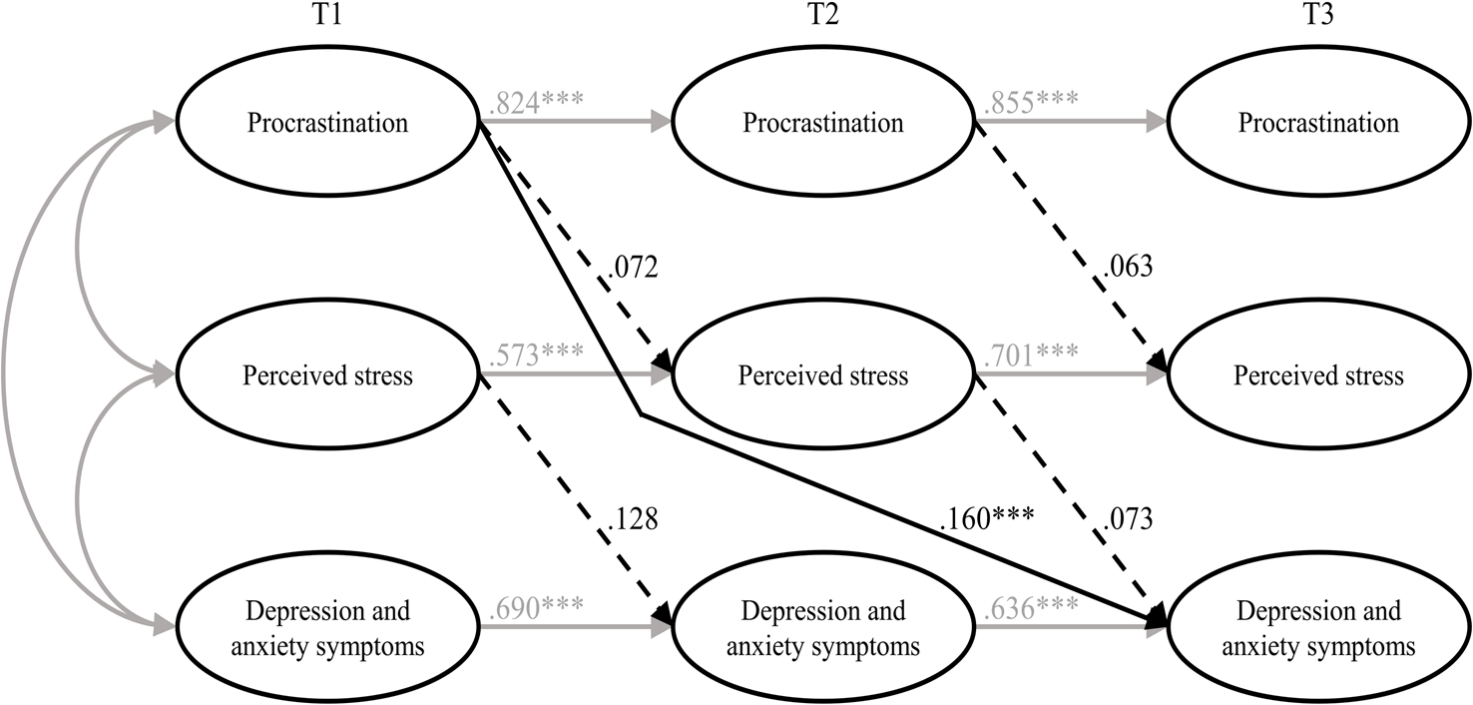
Results of the estimated model including all hypotheses (M_1_). *Note* Non-significant paths are dotted. T1 = time 1; T2 = time 2; T3 = time 3. *** *p* < .001

The autoregressive mediation model (M_1_) fitted the data significantly better than M_0_. The direct path from procrastination (T1) to depression and anxiety symptoms (T3) was significant (β = 0.16; *p* < .001), however, none of the mediated paths (from procrastination (T1) to perceived stress (T2) and from perceived stress (T2) to depression and anxiety symptoms (T3)) proved to be substantial. Also, the time-lagged paths from perceived stress (T1) to depression and anxiety symptoms (T2) and from procrastination (T2) to perceived stress (T3) were not substantial either (see Fig. 3).

To examine whether the hypothesized effects would occur over a one-year period rather than a six-months period, we specified an additional model with paths from procrastination (T1) to perceived stress (T3) and from perceived stress (T1) to depression and anxiety symptoms (T3), also including the stabilities of the three constructs as in the stability model M_0_. The model showed an acceptable fit (χ^2^ (486) = 831.281, *p* < .001; RMSEA = 0.048; SRMR = 0.091; TLI = 0.95; CFI = 0.95), but neither of the two paths were significant.

**Table 3:** Comparison of the measurement model and the structural models reflecting some of the key assumptions of the procrastination-health model over time

Model	x^2^ (df)	*p*	SRMR	RMSEA	TLI	CFI	AIC	Ref. Model	∆x^2^ (df)	*p*
M_M_	518.305 (429)	< 0.01	0.034	0.026	0.99	0.99	20,852.92			
M_0_	831.454 (486)	< 0.001	0.095	0.048	0.95	0.95	21,058.99			
M_1_	823.309 (482)	< 0.001	0.085	0.048	0.95	0.95	21,057.60	M_0_	7.98 (4)	> 0.05

*Note* M_M_ = measurement model, M_0_ = stability model, M_1_ = autoregressive mediation model; x^2^ = chi-square value; *df* = degrees of freedom; SRMR = standardized root mean square residual; RMSEA = root mean square error of approximation; TLI = Tucker-Lewis index; CFI = comparative fit index; AIC = Akaike information criterion; ∆x^2^ = Satorra-Bentler chi-square difference test [52]

Therefore, our hypotheses, that procrastination leads to perceived stress over time (H1) and that perceived stress leads to depression and anxiety symptoms over time (H2) must be rejected. We could only partially confirm our third hypothesis, that procrastination leads to depression and anxiety over time, mediated by perceived stress (H3), since procrastination did lead to depression and anxiety symptoms over time. However, this effect was not mediated by perceived stress.

## Discussion

To sum up, we tried to examine the harmful consequences of procrastination on students’ stress and mental health. Hence, we selected the procrastination-health model by Sirois [9] as a theoretical foundation and tried to evaluate some of its key assumptions in a temporal perspective. The author assumes that trait procrastination leads to (chronic) disease via (chronic) stress. We chose depression and anxiety symptoms as indicators for (chronic) disease and postulated, in line with the key assumptions of the procrastination-health model, that procrastination leads to perceived stress over time (H1), that perceived stress leads to depression and anxiety symptoms over time (H2), and that procrastination leads to depression and anxiety symptoms over time, mediated by perceived stress (H3). To examine these relationships properly, we collected longitudinal data from students at three occasions over a one-year period and analyzed the data using autoregressive time-lagged panel models. Our first and second hypotheses had to be rejected: Procrastination did not lead to perceived stress over time, and perceived stress did not lead to depression and anxiety symptoms over time. However, procrastination did lead to depression and anxiety symptoms over time – which is in line with our third hypothesis – but perceived stress was not a mediator of this effect. Therefore, we could only partially confirm our third hypothesis.

Our results contradict previous studies on the stress-related pathway of the procrastination-health model, which consistently found support for the role of (chronic) stress in the relationship between trait procrastination and (chronic) disease. Since most of these studies were cross-sectional, though, the causal direction of these effects remained uncertain. There are two longitudinal studies that confirm the stress-related pathway of the procrastination-health model [27, 28], but both studies examined short-term effects (≤ 3 months), whereas we focused on more long-term effects. Therefore, the divergent findings may indicate that there are short-term, but no long-term effects of trait procrastination on (chronic) disease mediated by (chronic) stress.

Our results especially raise the question whether trait procrastination leads to (chronic) stress in the long term. Looking at previous longitudinal studies on the effect of procrastination on stress, the following stands out: At shorter study periods of two weeks [27] and four weeks [28], the effect of procrastination on stress appears to be present. At longer study periods of seven weeks [59], three months [28], six months, and twelve months, as in our study, the effect of procrastination on stress does not appear to be present. There is one longitudinal study in which procrastination was a significant predictor of stress symptoms nine months later [34]. The results of this study should be interpreted with caution, though, because the outbreak of the COVID-19 pandemic fell within the study period, which could have contributed to increased stress symptoms [60]. Unfortunately, Johansson et al. [34] did not report whether average stress symptoms increased during their study. In one of the two studies conducted by Fincham and May [59], the COVID-19 pandemic outbreak also fell within their seven-week study period. However, they reported that in their study, average stress symptoms did not increase from baseline to follow-up. Taken together, the findings suggest that procrastination can cause acute stress in the short term, for example during times when many tasks need to be completed, such as at the end of a semester, but that procrastination does not lead to chronic stress over time. It seems possible that students are able to recover during the semester from the stress their procrastination caused at the end of the previous semester. Because of their procrastination, they may also have more time to engage in relaxing activities, which could further mitigate the effect of procrastination on stress. Our conclusions are supported by an early and well-known longitudinal study by Tice and Baumeister [61], which compared procrastinating and non-procrastinating students with regard to their health. They found that procrastinators experienced less stress than their non-procrastinating peers at the beginning of the semester, but more at the end of the semester. Additionally, our conclusions are in line with an interview study in which university students were asked about the consequences of their procrastination [62]. The students reported that, due to their procrastination, they experience high levels of stress during periods with heavy workloads (e.g., before deadlines or exams). However, the stress does not last, instead, it is relieved immediately after these periods.

Even though research indicates, in line with the assumptions of the procrastination-health model, that stress is a risk factor for physical and mental disorders [63–66], perceived stress did not have a significant effect on depression and anxiety symptoms in our study. The relationship between stress and mental health is complex, as people respond to stress in many different ways. While some develop stress-related mental disorders, others experience mild psychological symptoms or no symptoms at all [67]. This can be explained with the help of vulnerability-stress models. According to vulnerability-stress models, mental illnesses emerge from an interaction of vulnerabilities (e.g., genetic factors, difficult family backgrounds, or weak coping abilities) and stress (e.g., minor or major life events or daily hassles) [68, 69]. The stress perceived by the students in our sample may not be sufficient enough on its own, without the presence of other risk factors, to cause depression and anxiety symptoms. However, since we did not assess individual vulnerability and stress factors in our study, these considerations are mere speculation.

In our study, procrastination led to depression and anxiety symptoms over time, which is consistent with the procrastination-health model as well as previous cross-sectional and longitudinal evidence [18, 21, 31–34]. However, it is still unclear by which mechanisms this effect is mediated, as perceived stress did not prove to be a substantial mediator in our study. One possible mechanism would be that procrastination impairs affective well-being [70] and creates negative feelings, such as shame, guilt, regret, and anger [20–22, 62, 71], which in turn could lead to depression and anxiety symptoms [23–25]. Other potential mediators of the relationship between procrastination and depression and anxiety symptoms emerge from the behavioral pathway of the procrastination-health model, suggesting that poor health-related behaviors mediate the effect of trait procrastination on (chronic) disease. Although evidence for this is still scarce, the results of one cross-sectional study, for example, indicate that poor sleep quality might mediate the effect of procrastination on depression and anxiety symptoms [35].

In summary, we found that procrastination leads to depression and anxiety symptoms over time and that perceived stress is not a mediator of this effect. We could not show that procrastination leads to perceived stress over time, nor that perceived stress leads to depression and anxiety symptoms over time. For the most part, the relationships between procrastination, perceived stress, and depression and anxiety symptoms did not match the relationships between trait procrastination, (chronic) stress, and (chronic) disease as assumed in the procrastination-health model. Explanations for this could be that procrastination might only lead to perceived stress in the short term, for example, during preparations for end-of-semester exams, and that perceived stress may not be sufficient enough on its own, without the presence of other risk factors, to cause depression and anxiety symptoms. In conclusion, we could not confirm long-term effects of trait procrastination on (chronic) disease mediated by (chronic) stress, as assumed for the stress-related pathway of the procrastination-health model.

### Limitations and suggestions for future research

In our study, we tried to draw causal conclusions about the harmful consequences of procrastination on students’ stress and mental health. However, since procrastination is a trait that cannot be manipulated experimentally, we have conducted an observational rather than an experimental study, which makes causal inferences more difficult. Nonetheless, a major strength of our study is that we used a longitudinal design with three waves. This made it possible to draw conclusions about the causal direction of the effects, as in hardly any other study targeting consequences of procrastination on health before [4, 28, 55]. Therefore, we strongly recommend using a similar longitudinal design in future studies on the procrastination-health model or on consequences of procrastination on health in general.

We chose a time lag of six months between each of the three measurement occasions to examine long-term effects of procrastination on depression and anxiety symptoms mediated by perceived stress. However, more than six months may be necessary for the hypothesized effects to occur [72]. The fact that the temporal stabilities of the examined constructs were moderate or high (0.559 ≤ β ≤ 0.854) [73, 74] also suggests that the time lags may have been too short. The larger the time lag, the lower the temporal stabilities, as shown for depression and anxiety symptoms, for example [75]. High temporal stabilities make it more difficult to detect an effect that actually exists [76]. Nonetheless, Dormann and Griffin [77] recommend using shorter time lags of less than one year, even with high stabilities, because of other influential factors, such as unmeasured third variables. Therefore, our time lags of six months seem appropriate.

It should be discussed, though, whether it is possible to detect long-term effects of the stress-related pathway of the procrastination-health model within a total study period of one year. Sirois [9] distinguishes between short-term and long-term effects of procrastination on health mediated by stress, but does not address how long it might take for long-term effects to occur or when effects can be considered long-term instead of short-term. The fact that an effect of procrastination on stress is evident at shorter study periods of four weeks or less but in most cases not at longer study periods of seven weeks or more, as we mentioned earlier, could indicate that short-term effects occur within the time frame of one to three months, considering the entire stress-related pathway. Hence, it seems appropriate to assume that we have examined rather long-term effects, given our study period of six and twelve months. Nevertheless, it would be beneficial to use varying study periods in future studies, in order to be able to determine when effects can be considered long-term.

Concerning long-term effects of the stress-related pathway, Sirois [9] assumes that chronic procrastination causes chronic stress, which leads to chronic diseases over time. The term “chronic stress” refers to prolonged stress episodes associated with permanent tension. The instrument we used captures perceived stress over the last four weeks. Even though the perceived stress of the students in our sample was relatively stable (0.559 ≤ β ≤ 0.696), we do not know how much fluctuation occurred between each of the three occasions. However, there is some evidence suggesting that perceived stress is strongly associated with chronic stress [78]. Thus, it seems acceptable that we used perceived stress as an indicator for chronic stress in our study. For future studies, we still suggest the use of an instrument that can more accurately reflect chronic stress, for example, the Trier Inventory for Chronic Stress (TICS) [79].

It is also possible that the occasions were inconveniently chosen, as they all took place in a critical academic period near the end of the semester, just before the examination period began. We chose a similar period in the semester for each occasion for the sake of comparability. However, it is possible that, during this preparation periods, stress levels peaked and procrastinators procrastinated less because they had to catch up after delaying their work. This could have introduced bias to the data. Therefore, in future studies, investigation periods should be chosen that are closer to the beginning or in the middle of a semester.

Furthermore, Sirois [9] did not really explain her understanding of “chronic disease”. However, it seems clear that physical illnesses, such as diabetes or cardiovascular diseases, are meant. Depression and anxiety symptoms, which we chose as indicators for chronic disease, represent mental health complaints that do not have to be at the level of a major depressive disorder or an anxiety disorder, in terms of their quantity, intensity, or duration [40]. But they can be viewed as precursors to a major depressive disorder or an anxiety disorder. Therefore, given our study period of one year, it seems appropriate to use depression and anxiety symptoms as indicators for chronic disease. At longer study periods, we would expect these mental health complaints to manifest as mental disorders. Moreover, the procrastination-health model was originally designed to be applied to physical diseases [3]. Perhaps, the model assumptions are more applicable to physical diseases than to mental disorders. By applying parts of the model to mental health complaints, we have taken an important step towards finding out whether the model is applicable to mental disorders as well. Future studies should examine additional long-term health outcomes, both physical and psychological. This would help to determine whether trait procrastination has varying effects on different diseases over time. Furthermore, we suggest including individual vulnerability and stress factors in future studies in order to be able to analyze the effect of (chronic) stress on (chronic) diseases in a more differentiated way.

Regarding our sample, 3,420 students took part at the first occasion, but only 392 participated three times, which results in a dropout rate of 88.5%. At the second and third occasion, invitation e-mails were only sent to participants who had indicated at the previous occasion that they would be willing to participate in a repeat survey and provided their e-mail address. This is probably one of the main reasons for our high dropout rate. Other reasons could be that the students did not receive any incentives for participating in our study and that some may have graduated between the occasions. Selective dropout analysis revealed that the mean score of procrastination was lower in the group that participated in all three waves (*n* = 392) compared to the group that participated in the first wave (*n* = 3,420). One reason for this could be that those who have a higher tendency to procrastinate were more likely to procrastinate on filling out our survey at the second and third occasion. The findings of our dropout analysis should be kept in mind when interpreting our results, as lower levels of procrastination may have eliminated an effect on perceived stress or on depression and anxiety symptoms. Additionally, across all age groups in population-representative samples, the student age group reports having the best subjective health [80]. Therefore, it is possible that they are more resilient to stress and experience less impairment of well-being than other age groups. Hence, we recommend that future studies focus on other age groups as well.

## Conclusion

It is generally assumed that procrastination leads to lower academic performance, health impairment, and poor health-related behavior. However, evidence for negative consequences of procrastination is still limited and it is also unclear by which mechanisms they are mediated. In consequence, the aim of our study was to examine the effect of procrastination on mental health over time and stress as a possible facilitator of this relationship. We selected the procrastination-health model as a theoretical foundation and used the stress-related pathway of the model, assuming that trait procrastination leads to (chronic) disease via (chronic) stress. We chose depression and anxiety symptoms as indicators for (chronic) disease and collected longitudinal data from students at three occasions over a one-year period. This allowed us to draw conclusions about the causal direction of the effects, as in hardly any other study examining consequences of procrastination on (mental) health before. Our results indicate that procrastination leads to depression and anxiety symptoms over time and that perceived stress is not a mediator of this effect. We could not show that procrastination leads to perceived stress over time, nor that perceived stress leads to depression and anxiety symptoms over time. Explanations for this could be that procrastination might only lead to perceived stress in the short term, for example, during preparations for end-of-semester exams, and that perceived stress may not be sufficient on its own, that is, without the presence of other risk factors, to cause depression and anxiety symptoms. Overall, we could not confirm long-term effects of trait procrastination on (chronic) disease mediated by (chronic) stress, as assumed for the stress-related pathway of the procrastination-health model. Our study emphasizes the importance of identifying the consequences procrastination can have on health and well-being and determining by which mechanisms they are mediated. Only then will it be possible to develop interventions that can prevent negative health consequences of procrastination. Further health outcomes and possible mediators should be explored in future studies, using a similar longitudinal design.

### Electronic supplementary material

Below is the link to the electronic supplementary material.

**Supplementary Material 1** Selective dropout analysis and correlation matrix of the manifest variables

## Data Availability

The datasets used and/or analysed during the current study are available from the corresponding author on reasonable request.

## Footnotes

1 University Health Report at Freie Universität Berlin.

## Abbreviations

CFI: Comparative fit index
DSM-5: Diagnostic and Statistical Manual of Mental Disorders, Fifth Edition
GAD-2: Generalized Anxiety Disorder Scale-2
HEI-STRESS: Heidelberger Stress Index
HPA: Hypothalamic-pituitary-adrenocortical
MLR: Robust maximum likelihood estimation
PFS-4: Short form of the Procrastination Questionnaire for Students
PHQ-2: Patient Health Questionnaire-2
PHQ-4: Patient Health Questionnaire-4
RMSEA: Root mean square error of approximation
SEM: Structural equation modeling
SRMR: Standardized root mean square residual
TLI: Tucker-Lewis index
