# Supplementary material for: Procrastination, depression and anxiety symptoms in university students: a three-wave longitudinal study on the mediating role of perceived stress
Source: BMC Psychol. 2024 May 16;12:276. doi: 10.1186/s40359-024-01761-2 (PMC11100206; doi:10.1186/s40359-024-01761-2)
Supplement: Supplementary file 1 — Supplementary Material 1 Selective dropout analysis and correlation matrix of the manifest variables [file 40359_2024_1761_MOESM1_ESM.pdf]

Supplementary material

Table S1 Selective dropout analysis.

Variables/Scales	*M* Participants of first wave (n = 3,420)	*M* Participants of third wave (n = 392)	t	df	p
Age T1	24.06	24.08	-0.08	3415	> .05
Academic semester T1	7.09	7.04	0.20	3399	> .05
Number of assessments/exams T1	2.63	2.67	-0.31	3328	> .05
Procrastination T1	3.27	3.15	2.08	3409	< .05
Perceived stress T1	65.16	64.25	0.82	3328	> .05
Depression and anxiety symptoms T1	4.92	4.83	0.50	3345	> .05

Note. t = t value; df = degrees of freedom; T1 = time 1.

Table S2 Selective dropout analysis.

Gender	% Participants of first wave (n = 3,420)	% Participants of third wave (n = 392)	χ^2	df	p
Female	71.53 (n = 2,166)	73.98 (n = 290)			
Male	26.98 (n = 817)	24.23 (n = 95)			
Other	1.49 (n = 45)	1.79 (n = 7)			
			1.48	2	> .05

Note. χ^2 = chi-square value; df = degrees of freedom.

Table S3 Correlation matrix of the manifest variables.

	Item		1	2	3	4	5	6	7	8	9	10	11	12	13	14	15	16	17	18	19	20	21	22	23	24	25	26	27	28	29	30	31	32	33
1	PRO1	T1	1																																
2	PRO2	T1	.76	1																															
3	PRO3	T1	.67	.62	1																														
4	PRO4	T1	.79	.71	.67	1																													
5	PRO1	T2	.71	.60	.52	.62	1																												
6	PRO2	T2	.61	.62	.49	.53	.72	1																											
7	PRO3	T2	.57	.53	.56	.50	.69	.65	1																										
8	PRO4	T2	.65	.62	.51	.64	.83	.74	.70	1																									
9	PRO1	T3	.71	.61	.51	.66	.70	.60	.57	.71	1																								
10	PRO2	T3	.65	.60	.47	.59	.61	.63	.55	.64	.83	1																							
11	PRO3	T3	.56	.57	.57	.59	.58	.55	.62	.58	.68	.70	1																						
12	PRO4	T3	.66	.60	.53	.69	.69	.60	.58	.70	.84	.81	.73	1																					
13	PS1	T1	-.04	-.06	-.01	-.09	-.06	-.02	-.01	-.07	.00	.03	-.02	-.02	1																				
14	PS2	T1	.08	.11	.05	.02	.03	.09	.05	.06	.10	.13	.03	.06	.68	1																			
15	PS3	T1	.04	-.02	-.01	.00	-.03	-.02	.02	-.02	.03	.06	-.03	.02	.68	.66	1																		
16	PS1	T2	.09	.06	.10	.03	.05	.03	.02	.01	.08	.14	.08	.09	.41	.38	.32	1																	
17	PS2	T2	.09	.04	.02	.02	.10	.16	.09	.08	.12	.18	.07	.11	.43	.49	.44	.63	1																
18	PS3	T2	.09	.04	.01	.07	.06	.04	.01	.02	.08	.09	.02	.04	.32	.38	.37	.64	.70	1															
19	PS1	T3	.18	.10	.15	.04	.13	.14	.11	.10	.21	.26	.15	.14	.39	.36	.35	.45	.41	.33	1														
20	PS2	T3	.14	.12	.06	.08	.10	.16	.06	.10	.21	.26	.11	.14	.40	.56	.42	.45	.58	.49	.59	1													
21	PS3	T3	.08	.02	.07	-.01	.01	.06	.02	-.02	.08	.14	.01	.04	.38	.44	.44	.45	.57	.52	.64	.69	1												
22	DAS1	T1	.21	.23	.15	.19	.17	.25	.09	.19	.21	.22	.14	.23	.34	.49	.38	.20	.28	.22	.24	.39	.25	1											
23	DAS2	T1	.17	.17	.14	.12	.15	.21	.13	.15	.17	.19	.16	.18	.37	.51	.37	.24	.33	.27	.22	.39	.27	.64	1										
24	DAS3	T1	.18	.16	.11	.10	.13	.19	.09	.12	.17	.19	.12	.14	.44	.58	.40	.27	.35	.30	.28	.40	.29	.46	.60	1									
25	DAS4	T1	.22	.19	.15	.18	.18	.24	.17	.20	.23	.24	.14	.23	.37	.53	.38	.23	.34	.25	.27	.45	.30	.56	.72	.75	1								
26	DAS1	T2	.16	.18	.04	.10	.17	.23	.08	.17	.18	.21	.13	.19	.34	.45	.33	.38	.46	.37	.29	.42	.34	.56	.52	.37	.44	1							
27	DAS2	T2	.21	.19	.08	.16	.21	.28	.15	.23	.23	.24	.15	.24	.34	.46	.33	.41	.50	.38	.27	.43	.34	.50	.57	.45	.51	.68	1						
28	DAS3	T2	.10	.04	.00	.06	.10	.10	.05	.09	.13	.16	.04	.11	.38	.53	.36	.46	.62	.50	.34	.53	.44	.39	.43	.52	.48	.56	.63	1					
29	DAS4	T2	.10	.11	.01	.04	.08	.14	.04	.11	.13	.13	.03	.09	.32	.50	.31	.38	.49	.38	.25	.45	.31	.39	.53	.51	.60	.60	.73	.65	1				
30	DAS1	T3	.23	.20	.14	.19	.22	.26	.16	.23	.33	.34	.26	.28	.25	.33	.24	.28	.33	.29	.40	.54	.41	.46	.42	.37	.41	.53	.43	.40	.37	1			
31	DAS2	T3	.31	.23	.19	.22	.27	.28	.22	.27	.33	.34	.27	.30	.21	.37	.28	.31	.37	.27	.40	.52	.42	.44	.49	.37	.45	.48	.55	.48	.46	.68	1		
32	DAS3	T3	.18	.11	.13	.10	.17	.17	.07	.16	.23	.21	.12	.15	.31	.46	.35	.28	.38	.33	.42	.59	.49	.38	.43	.52	.48	.44	.44	.60	.45	.62	.65	1	
33	DAS4	T3	.16	.15	.10	.11	.14	.22	.12	.18	.24	.27	.19	.20	.28	.43	.30	.25	.37	.26	.42	.54	.43	.38	.53	.53	.60	.48	.54	.49	.56	.61	.71	.66	1

Note. PRO = Procrastination; PS = Perceived stress; DAS = Depression and anxiety symptoms; T1 = time 1; T2 = time 2; T3 = time 3.
